# Supplementary material for: The Effectiveness of Molecular, Karyotype and Morphological Methods in the Identification of Morphologically Conservative Sibling Species: An Integrative Taxonomic Case of the Crocidura attenuata Species Complex in Mainland China
Source: Animals (Basel). 2023 Feb 12;13(4):643. doi: 10.3390/ani13040643 (PMC9951653; doi:10.3390/ani13040643)
Supplement: Supplementary file 1 [file animals-13-00643-s001.zip › Table S1.pdf]

Table S1. Sample information of Chinese *Crocidura attenuata* species complex used in Current study

Species	Specimen Label	GenBank Number	Haplotype	karyotype (2n, FN)	Morphological analysis	Sample site in Fig.1	Sample Location	Reference to *Cytb*	Reference to karyotype
C. attenuata	S1608	OP594738	78			1	Baoxing, Sichuan	Current study	
C. attenuata	S1610	MK765715	79		Yes	1	Baoxing, Sichuan	[30]	
C. attenuata	S1611	MK765716	78			1	Baoxing, Sichuan	[30]	
C. attenuata	S1615	MK765717	80			1	Baoxing, Sichuan	[30]	
C. attenuata	S1655	MK765718	81			1	Baoxing, Sichuan	[30]	
C. attenuata	S1657	OP594739	81			1	Baoxing, Sichuan	Current study	
C. attenuata	S1658	OP594740	81		Yes	1	Baoxing, Sichuan	Current study	
C. attenuata	S1659	OP594741	81			1	Baoxing, Sichuan	Current study	
C. attenuata	S2014	MK765719	81		Yes	1	Baoxing, Sichuan	[30]	
C. attenuata	S2051	MK765720	81		Yes	1	Baoxing, Sichuan	[30]	
C. attenuata	S2216	MK765721	82		Yes	3	Shennongjia, Hubei	[30]	
C. attenuata	S2226	MK765722	82		Yes	3	Shennongjia, Hubei	[30]	
C. attenuata	S2537	MK765730	83			1	Baoxing, Sichuan	[30]	
C. attenuata	S2540	MK765731	84		Yes	1	Baoxing, Sichuan	[30]	
C. attenuata	S2541	OP594742	83		Yes	1	Baoxing, Sichuan	Current study	
C. attenuata	S2559	MK978258	83	40, 54	Yes	1	Baoxing, Sichuan	[34]	[34]
C. attenuata	S2565	MK765732	83			1	Baoxing, Sichuan	[30]	
C. attenuata	S2567	MK765734	84			1	Baoxing, Sichuan	[30]	
C. attenuata	S2570	MK978259	84	40, 54	Yes	1	Baoxing, Sichuan	[34]	[34]

C. attenuata	S2572	MK978260	83	40, 54		1	Baoxing, Sichuan	[34]	[34]
C. attenuata	S2576	MK765735	83			1	Baoxing, Sichuan	[30]	
C. attenuata	S2577	MK765736	85		Yes	1	Baoxing, Sichuan	[30]	
C. attenuata	S2665	OP594743	78			1	Baoxing, Sichuan	Current study	
C. attenuata	S2670	MK765737	79			1	Baoxing, Sichuan	[30]	
C. attenuata	S2682	MK765738	78			1	Baoxing, Sichuan	[30]	
C. attenuata	S2684	MK978261	86	40, 54		1	Baoxing, Sichuan	[34]	[34]
C. attenuata	S2699	OP594744	87			1	Baoxing, Sichuan	Current study	
C. attenuata	S2717	MK765739	81			1	Baoxing, Sichuan	[30]	
C. attenuata	S2720	MK765740	81			1	Baoxing, Sichuan	[30]	
C. attenuata	S2739	MK978262	80	40, 54		1	Baoxing, Sichuan	[34]	[34]
C. attenuata	S2740	MK978263	81	40, 54	Yes	1	Baoxing, Sichuan	[34]	[34]
C. attenuata	S2745	OP594745	81			1	Baoxing, Sichuan	Current study	
C. attenuata	S2753	OP594746	87			1	Baoxing, Sichuan	Current study	
C. attenuata	S2758	OP594747	81			1	Baoxing, Sichuan	Current study	
C. attenuata	S2760	OP594748	81			1	Baoxing, Sichuan	Current study	
C. attenuata	S2765	OP594749	81		Yes	1	Baoxing, Sichuan	Current study	
C. attenuata	S2766	MK978264	80	40, 54	Yes	1	Baoxing, Sichuan	[34]	[34]
C. attenuata	S2767	MK765741	81			1	Baoxing, Sichuan	[30]	
C. attenuata	S2770	MK765742	87			1	Baoxing, Sichuan	[30]	
C. attenuata	S2771	MK765743	81			1	Baoxing, Sichuan	[30]	

C. attenuata	S2772	MK765744	81			1	Baoxing, Sichuan	[30]	
C. attenuata	S2798	MK765745	80			1	Baoxing, Sichuan	[30]	
C. attenuata	S2800	MK765746	87			1	Baoxing, Sichuan	[30]	
C. attenuata	S2801	MK765747	80			1	Baoxing, Sichuan	[30]	
C. attenuata	S2802	MK765748	81			1	Baoxing, Sichuan	[30]	
C. attenuata	S2831	MK978265	81	40, 54	Yes	1	Baoxing, Sichuan	[34]	[34]
C. attenuata	S2833	OP594750	80			1	Baoxing, Sichuan	Current study	
C. attenuata	S2841	OP594751	87			1	Baoxing, Sichuan	Current study	
C. attenuata	S4740	OP594752	88		Yes	2	Ningqiang, Shaanxi	Current study	
C. attenuata	S4796	OP594753	81			1	Baoxing, Sichuan	Current study	
C. attenuata	S4806	OP594754	81			1	Baoxing, Sichuan	Current study	
C. attenuata	S4814	OP594755	81			1	Baoxing, Sichuan	Current study	
C. attenuata	S4815	OP594756	80			1	Baoxing, Sichuan	Current study	
C. attenuata		MN690999	94			A5	Qingchuan, Sichuan	[16]	
C. attenuata		MN691000	91			A5	Qingchuan, Sichuan	[16]	
C. attenuata		MN691001	91			A5	Qingchuan, Sichuan	[16]	
C. attenuata		MN691002	92			A5	Qingchuan, Sichuan	[16]	
C. attenuata		MN691003	93			A4	Pengzhou, Sichuan	[16]	
C. attenuata		MN691004	90			A1	Mingshan, Sichuan	[16]	
C. attenuata		MN691005	95			A1	Mingshan, Sichuan	[16]	
C. attenuata		MN691006	95			A1	Mingshan, Sichuan	[16]	

C. attenuata		MN691007	95			A2	Yingjing, Sichuan	[16]	
C. attenuata		MN691008	95			A2	Yingjing, Sichuan	[16]	
C. attenuata		MN691009	95			A2	Yingjing, Sichuan	[16]	
C. attenuata		MN691010	96			A5	Qingchuan, Sichuan	[16]	
C. attenuata		MN691011	95			A2	Yingjing, Sichuan	[16]	
C. attenuata		MN691012	91			A5	Qingchuan, Sichuan	[16]	
C. attenuata		MN691013	98			A5	Qingchuan, Sichuan	[16]	
C. attenuata		MN691014	99			A3	Muchuan, Sichuan	[16]	
C. attenuata		MN691015	89			A6	Kaixian, Chongqing	[16]	
C. attenuata		MN691016	97			A7	Mt. Huaying, Sichuan	[16]	
C. anhuiensis	G09201	MK765684	102	40, 54		8	Nanling, Guangdong	[30]	[34]
C. anhuiensis	G09203	MK765685	102	40, 54	Yes	8	Nanling, Guangdong	[30]	[34]
C. anhuiensis	S1057	MK765708	103			6	Mt. Wuyishan, Fujian	[30]	
C. anhuiensis	S1071	OP594757	104			5	Mt. Wuyishan, Jiangxi	Current study	
C. anhuiensis	S1414	MK765713	105		Yes	4	Jinhua, Zhejiang	[30]	
C. anhuiensis	S1458	MK765714	106		Yes	4	Jinhua, Zhejiang	[30]	
C. anhuiensis	S3358	MK765763	105	40, 54	Yes	4	Jinhua, Zhejiang	[30]	[34]
C. anhuiensis	S3726	MK765768	107			7	Jinggangshan, Jiangxi	[30]	
C. anhuiensis	S4183	OP594758	106		Yes	4	Jinhua, Zhejiang	Current study	
C. anhuiensis	S4188	OP594759	106		Yes	4	Jinhua, Zhejiang	Current study	
C. anhuiensis	S4214	OP594760	106		Yes	4	Jinhua, Zhejiang	Current study	
C. anhuiensis	S4342	OP594761	108		Yes	4	Jinhua, Zhejiang	Current study	

C. anhuiensis		MK546383	109			B1	Mt. Huang, Anhui	[31]	
C. anhuiensis		MK546384	110			B1	Mt. Huang, Anhui	[31]	
C. anhuiensis		MK546385	110			B1	Mt. Huang, Anhui	[31]	
C. anhuiensis		MK546386	111			B1	Mt. Huang, Anhui	[31]	
C. anhuiensis		MK546387	112			B1	Mt. Huang, Anhui	[31]	
C. anhuiensis		MK546388	112			B1	Mt. Huang, Anhui	[31]	
C. anhuiensis		FJ814034	100			B2	Yongzhou, Hunan	[26]	
C. dongyangjiangensis	G09202	OP594762	113		Yes	8	Nanling, Guangdong	Current study	
C. dongyangjiangensis	G09247	OP594763	114	40, 54	Yes	8	Nanling, Guangdong	Current study	Current study
C. dongyangjiangensis	G09248	OP594764	115	40, 54	Yes	8	Nanling, Guangdong	Current study	Current study
C. dongyangjiangensis	G09322	OP594765	116		Yes	9	Yingde, Guangdong	Current study	
C. dongyangjiangensis	G12189	OP594766	117	40, 54	Yes	8	Nanling, Guangdong	Current study	Current study
C. dongyangjiangensis	S0440	OP594767	118		Yes	7	Suichuan, Jiangxi	Current study	
C. dongyangjiangensis	S0962	OP594768	119			5	Mt. Wuyishan, Jiangxi	Current study	
C. dongyangjiangensis	S1413	OP594769	120	40, 54	Yes	4	Jinhua, Zhejiang	Current study	Current study
C. dongyangjiangensis	S1431	OP594770	121	40, 54	Yes	4	Jinhua, Zhejiang	Current study	Current study
C. dongyangjiangensis	S3924	OP594771	121		Yes	4	Jinhua, Zhejiang	Current study	
C. dongyangjiangensis	S3989	OP594772	121		Yes	4	Jinhua, Zhejiang	Current study	

C. dongyangjiangensis	S3990	OP594773	120		Yes	4	Jinhua, Zhejiang	Current study	
C. dongyangjiangensis	S4343	OP594774	121			4	Jinhua, Zhejiang	Current study	
C. dongyangjiangensis		MN364684	129			B1	Mt. Huang, Anhui	[32]	
C. dongyangjiangensis		MN364685	130			B1	Mt. Huang, Anhui	[32]	
C. dongyangjiangensis		MN364686	131			B1	Mt. Huang, Anhui	[32]	
C. dongyangjiangensis		MN364687	132			B1	Mt. Huang, Anhui	[32]	
C. dongyangjiangensis		MN364688	131			B1	Mt. Huang, Anhui	[32]	
C. dongyangjiangensis		MK797049	124			C1/C2	Dongyang, Zhejiang, Quzhou, Zhejiang	[49]	
C. dongyangjiangensis		MK797050	123			C1/C2	Dongyang, Zhejiang, Quzhou, Zhejiang	[49]	
C. dongyangjiangensis		MK797051	127			C1/C2	Dongyang, Zhejiang, Quzhou, Zhejiang	[49]	
C. dongyangjiangensis		MN691018	122			C3	Hongjiang, Hunan	[16]	
C. dongyangjiangensis		MN691019	122			C3	Hongjiang, Hunan	[16]	
C. dongyangjiangensis		MN691020	123			C2	Dongyang, Zhejiang	[16]	
C. dongyangjiangensis		MN691021	124			C2	Dongyang, Zhejiang	[16]	
C. dongyangjiangensis		MN691022	125			C2	Dongyang, Zhejiang	[16]	
C. dongyangjiangensis		MN691023	126			C2	Dongyang, Zhejiang	[16]	
C. dongyangjiangensis		MN691024	124			C2	Dongyang, Zhejiang	[16]	
C. dongyangjiangensis		MN691025	124			C2	Dongyang, Zhejiang	[16]	
C. dongyangjiangensis		MN691026	127			C2	Dongyang, Zhejiang	[16]	
C. dongyangjiangensis		MN691027	128			C2	Dongyang, Zhejiang	[16]	
C. tanakae	G09193	MK765682	1	26, 48	Yes	8	Nanling, Guangdong	[30]	[34]
C. tanakae	G09196	MK765683	2			8	Nanling, Guangdong	[30]	

C. tanakae	G09266	MK765686	3	26, 48		8	Mt. Nanling, Guangdong	[30]	[34]
C. tanakae	G09286	MK978266	4	38, 54		9	Yingde, Guangdong	[34]	[34]
C. tanakae	G09310	MK978267	5	27, 48		9	Yingde, Guangdong	[34]	[34]
C. tanakae	G09311	OP594775	6			9	Yingde, Guangdong	Current study	
C. tanakae	G09312	MK978268	7	27, 48		9	Yingde, Guangdong	[34]	[34]
C. tanakae	G09313	MK978269	8	26, 46		9	Yingde, Guangdong	[34]	[34]
C. tanakae	G09314	MK978270	9	28, 48		9	Yingde, Guangdong	[34]	[34]
C. tanakae	G09315	MK765687	10			9	Yingde, Guangdong	[30]	
C. tanakae	G09316	MK765688	11			9	Yingde, Guangdong	[30]	
C. tanakae	G09317	MK765689	12			9	Yingde, Guangdong	[30]	
C. tanakae	G09318	MK978271	13	27, 47	Yes	9	Yingde, Guangdong	[34]	[34]
C. tanakae	G09319	MK978272	8	27, 47		9	Yingde, Guangdong	[34]	[34]
C. tanakae	G09320	MK765690	14			9	Yingde, Guangdong	[30]	
C. tanakae	G09321	MK978273	9	27, 48		9	Yingde, Guangdong	[34]	[34]
C. tanakae	G09323	MK978274	4	28, 47		9	Yingde, Guangdong	[34]	[34]
C. tanakae	G09325	MK765691	15	27, 47	Yes	9	Yingde, Guangdong	[30]	[34]
C. tanakae	G12111	MK978275	16	34, 52		12	Qiongzhong, Hainan	[34]	[34]
C. tanakae	G12328	MK765692	4			8	Luokeng, Shaoguan, Guangdong	[30]	
C. tanakae	G12349	MK978276	17	29, 48		8	Luokeng, Shaoguan, Guangdong	[34]	[34]
C. tanakae	G12350	MK765693	17	26, 48	Yes	8	Luokeng, Shaoguan, Guangdong	[34]	[34]
C. tanakae	G12417	MK765694	18	40, 56		10	Shenzhen, Guangdong	[34]	[34]
C. tanakae	M04496	MK765695	19		Yes	26	Mt. Emei, Sichuan	[30]	
C. tanakae	M04497	MK978277	20			26	Mt. Emei, Sichuan	[34]	

C. tanakae	M04505	MK765696	21		Yes	13	Mt. Diaoluoshan, Hainan	[30]	
C. tanakae	MH8237	MK978278	9	28, 47		9	Yingde, Guangdong	[34]	[34]
C. tanakae	MH8238	MK765697	9	28, 47		9	Yingde, Guangdong	[30]	[34]
C. tanakae	MH8239	MK978279	8	29, 48		9	Yingde, Guangdong	[34]	[34]
C. tanakae	MH8253	MK765698	4			9	Yingde, Guangdong	[30]	14
C. tanakae	MH8254	MK765699	4	37, 55	Yes	9	Yingde, Guangdong	[30]	[34]
C. tanakae	MH8255	MK978280	4	38, 53		9	Yingde, Guangdong	[34]	[34]
C. tanakae	MH8256	MK978281	4	36, 53		9	Yingde, Guangdong	[34]	[34]
C. tanakae	MH8257	MK978282	22	37, 55		9	Yingde, Guangdong	[34]	[34]
C. tanakae	MH8258	MK978283	4	37, 54		9	Yingde, Guangdong	[34]	[34]
C. tanakae	MH8259	MK978284	23	35, 52		9	Yingde, Guangdong	[34]	[34]
C. tanakae	MH8260	MK978285	22	38, 55	Yes	9	Yingde, Guangdong	[34]	[34]
C. tanakae	MH8261	MK978286	4	38, 54	Yes	9	Yingde, Guangdong	[34]	[34]
C. tanakae	MH8283	MK765791	4	36, 54		10	Longmen, Guangdong	[30]	[34]
C. tanakae	MH8284	MK978287	24	38, 55		10	Longmen, Guangdong	[34]	[34]
C. tanakae	MH8285	MK765700	4	36, 54		10	Longmen, Guangdong	[30]	[34]
C. tanakae	MH8286	MK765701	4	35, 55		10	Longmen, Guangdong	[30]	[34]
C. tanakae	MH8287	MK765702	4	37, 55		10	Longmen, Guangdong	[30]	[34]
C. tanakae	MH8290	MK765703	25	37, 55		11	Guangzhou, Guangdong	[30]	[34]
C. tanakae	MH8291	MK765704	4	40, 56		11	Guangzhou, Guangdong	[30]	[34]
C. tanakae	MH8292	MK978288	4	38, 56	Yes	11	Guangzhou, Guangdong	[34]	[34]
C. tanakae	MH8293	MK978289	26	40, 56		11	Guangzhou, Guangdong	[34]	[34]
C. tanakae	MH8294	MK978290	4	39, 56		11	Guangzhou, Guangdong	[34]	[34]
C. tanakae	MH8414	MK978291	27	36, Uncertain		26	Mt. Emei, Sichuan	[34]	[34]
C. tanakae	MH8415	MK978292	27	37, 54	Yes	26	Mt. Emei, Sichuan	[34]	[34]

C. tanakae	MH8416	MK765704	27	35, 53		26	Mt. Emei, Sichuan	[30]	[34]
C. tanakae	MH8417	MK978293	28	36, 53		26	Mt. Emei, Sichuan	[34]	[34]
C. tanakae	S0325	MK978294	29	24, 46	Yes	20	Beiliu, Guangxi	[34]	[34]
C. tanakae	S0329	MK978295	30	25, 46		20	Beiliu, Guangxi	[34]	[34]
C. tanakae	S0702	OP594776	31			18	Jishou, Hunan	Current study	
C. tanakae	S0703	MK765706	6			18	Jishou, Hunan	[30]	
C. tanakae	S0731	MK765707	6			18	Jishou, Hunan	[30]	
C. tanakae	S0732	OP594777	6		Yes	18	Jishou, Hunan	Current study	
C. tanakae	S1364	OP594778	32			21	Chongzuo, Guangxi	Current study	
C. tanakae	S1369	OP594779	33			21	Chongzuo, Guangxi	Current study	
C. tanakae	S1370	MK765709	33			21	Chongzuo, Guangxi	[30]	
C. tanakae	S1371	MK765710	33			21	Chongzuo, Guangxi	[30]	
C. tanakae	S1385	MK765711	33			21	Longzhou, Guangxi	[30]	
C. tanakae	S1404	MK765712	33			21	Longzhou, Guangxi	[30]	
C. tanakae	S1423	OP594780	34			4	Jinhua, Zhejiang	Current study	
C. tanakae	S1424	OP594781	34			4	Jinhua, Zhejiang	Current study	
C. tanakae	S1438	OP594782	34			4	Jinhua, Zhejiang	Current study	
C. tanakae	S1454	OP594783	34			4	Jinhua, Zhejiang	Current study	
C. tanakae	S2421	OP594784	35			14	Mt. Dabieshan, Anhui	Current study	

C. tanakae	S2426	MK765723	36		Yes	14	Mt. Dabieshan, Anhui	[30]	
C. tanakae	S2432	MK978296	37	37, Uncertain		14	Mt. Dabieshan, Anhui	[34]	[34]
C. tanakae	S2438	MK765724	38		Yes	14	Mt. Dabieshan, Anhui	[30]	
C. tanakae	S2442	OP594785	36			15	Mt. Shuangfengshan, Xiaogan, Hubei	Current study	
C. tanakae	S2444	MK978297	36	37, 54		15	Mt. Shuangfengshan, Xiaogan, Hubei	[34]	[34]
C. tanakae	S2468	MK765725	36		Yes	15	Mt. Shuangfengshan, Xiaogan, Hubei	[30]	
C. tanakae	S2475	MK765726	36		Yes	15	Mt. Shuangfengshan, Xiaogan, Hubei	[30]	
C. tanakae	S2477	MK765727	31			17	Zhangjiajie, Hunan	[30]	
C. tanakae	S2485	OP594786	31			17	Zhangjiajie, Hunan	Current study	
C. tanakae	S2501	MK765728	39		Yes	23	Mt. Wulingshan, Chongqing	[30]	
C. tanakae	S2523	MK765729	40			23	Mt. Wulingshan, Chongqing	[30]	
C. tanakae	S2566	MK765733	41		Yes	1	Baoxing, Sichuan	[30]	
C. tanakae	S2869	MK765749	42			1	Baoxing, Sichuan	[30]	
C. tanakae	S2930	MK765750	43		Yes	27	Luoping, Yunnan	[30]	
C. tanakae	S3089	OP594787	44		Yes	19	Mt. Mangshan, Chenzhou, Hunan	Current study	
C. tanakae	S3114	MK765751	4			42	Ningdu, Jiangxi	[30]	
C. tanakae	S3155	MK765752	4			6	Mt. Wuyishan, Fujian	[30]	
C. tanakae	S3185	MK765753	4			6	Mt. Wuyishan, Fujian	[30]	
C. tanakae	S3186	MK765754	4			6	Mt. Wuyishan, Fujian	[30]	

C. tanakae	S3263	MK765755	45	39, 56		5	Qianshan,Jiangxi	[30]	[34]
C. tanakae	S3272	MK765756	34			4	Jinhua, Zhejiang	[30]	
C. tanakae	S3273	MK765757	46			4	Jinhua, Zhejiang	[30]	
C. tanakae	S3318	MK765758	34			4	Jinhua, Zhejiang	[30]	
C. tanakae	S3338	MK765759	46	36, 52		4	Jinhua, Zhejiang	[30]	[34]
C. tanakae	S3339	MK978298	34	39, 54		4	Jinhua, Zhejiang	[34]	[34]
C. tanakae	S3340	MK978299	34	38, 53		4	Jinhua, Zhejiang	[34]	[34]
C. tanakae	S3341	MK978300	34	37, 55		4	Jinhua, Zhejiang	[34]	[34]
C. tanakae	S3345	MK765762	17		Yes	4	Jinhua, Zhejiang	[30]	
C. tanakae	S3357	MK978301	34	37, 52		4	Jinhua, Zhejiang	[34]	[34]
C. tanakae	S3359	MK978302	34	37, 55		4	Jinhua, Zhejiang	[34]	[34]
C. tanakae	S3362	OP594788	34			4	Jinhua, Zhejiang	Current study	
C. tanakae	S3388	MK978303	46	39, Uncertain		4	Jinhua, Zhejiang	[34]	[34]
C. tanakae	S3505	MK978304	4	38, 56		6	Mt. Wuyishan, Fujian	[34]	[34]
C. tanakae	S3507	MK978305	4	39, 56		6	Mt. Wuyishan, Fujian	[34]	[34]
C. tanakae	S3508	MK765764	4		Yes	6	Mt. Wuyishan, Fujian	[30]	14
C. tanakae	S3540	MK978306	4	39, 56		6	Mt. Wuyishan, Fujian	[34]	[34]
C. tanakae	S3552	MK765765	17	36, 54	Yes	7	Jinggangshan, Jiangxi	[30]	[34]
C. tanakae	S3624	MK765766	9	28, 45		9	Yingde, Guangdong	[30]	[34]
C. tanakae	S3654	MK765767	47	34, 52	Yes	12	Qiongzhong, Hainan	[30]	[34]
C. tanakae	S3737	MK765769	17	36, 52		7	Jinggangshan, Jiangxi	[30]	[34]
C. tanakae	S3772	MK765761	17	36, Uncertain		7	Jinggangshan, Jiangxi	[30]	[34]
C. tanakae	S3773	MK765760	17	38, Uncertain		7	Jinggangshan, Jiangxi	[30]	[34]

C. tanakae	S3774	MK765770	17	37, Uncertain		7	Jinggangshan, Jiangxi	[30]	[34]
C. tanakae	S3775	MK978307	48	38, 55		7	Jinggangshan, Jiangxi	[34]	[34]
C. tanakae	S3782	MK978308	17	38, 54	Yes	7	Jinggangshan, Jiangxi	[34]	[34]
C. tanakae	S3783	MK978309	49	39, Uncertain		7	Jinggangshan, Jiangxi	[34]	[34]
C. tanakae	S3840	MK978310	45	39, 56	Yes	5	Qianshan,Jiangxi	[34]	[34]
C. tanakae	S3841	MK978311	34	38, 56		5	Qianshan,Jiangxi	[34]	[34]
C. tanakae	S3845	MK765771	34			5	Qianshan,Jiangxi	[30]	
C. tanakae	S3846	MK765772	34			5	Qianshan,Jiangxi	[30]	
C. tanakae	S3847	MK765773	34			5	Qianshan,Jiangxi	[30]	
C. tanakae	S3850	MK765774	34			5	Qianshan,Jiangxi	[30]	
C. tanakae	S3853	MK765775	17			5	Qianshan,Jiangxi	[30]	
C. tanakae	S3867	MK978312	4	39, 54		5	Qianshan,Jiangxi	[34]	[34]
C. tanakae	S3868	MK978313	4	39, 54		5	Qianshan,Jiangxi	[34]	[34]
C. tanakae	S3869	MK978314	17	39, 56		5	Qianshan,Jiangxi	[34]	[34]
C. tanakae	S3870	MK978315	34	40, 56		5	Qianshan,Jiangxi	[34]	[34]
C. tanakae	S3880	MK978316	45	40, 56	Yes	5	Qianshan,Jiangxi	[34]	[34]
C. tanakae	S3881	MK978317	45	38, 54		5	Qianshan,Jiangxi	[34]	[34]
C. tanakae	S3920	MK765776	34	38, 54		4	Jinhua, Zhejiang	[30]	[34]
C. tanakae	S3921	MK765777	17	37, 55	Yes	4	Jinhua, Zhejiang	[30]	[34]
C. tanakae	S3922	MK765778	17	37, 55		4	Jinhua, Zhejiang	[30]	[34]
C. tanakae	S3954	MK765779	17			4	Jinhua, Zhejiang	[30]	
C. tanakae	S3957	MK978318	46	39, 56		4	Jinhua, Zhejiang	[34]	[34]
C. tanakae	S3958	MK978319	34	38, 54		4	Jinhua, Zhejiang	[34]	[34]
C. tanakae	S3959	MK978320	34	39, 56		4	Jinhua, Zhejiang	[34]	[34]

C. tanakae	S3960	MK765780	34	37, 55		4	Jinhua, Zhejiang	[30]	[34]
C. tanakae	S3984	MK765781	34			4	Jinhua, Zhejiang	[30]	
C. tanakae	S3987	MK765782	50	39, 54		4	Jinhua, Zhejiang	[30]	[34]
C. tanakae	S3988	MK765783	17	39, 56		4	Jinhua, Zhejiang	[30]	[34]
C. tanakae	S4011	MK765784	17	39, Uncertain		4	Jinhua, Zhejiang	[30]	[34]
C. tanakae	S4012	MK765785	17	39, 56		4	Jinhua, Zhejiang	[30]	[34]
C. tanakae	S4013	MK765786	34	37, 54		4	Jinhua, Zhejiang	[30]	[34]
C. tanakae	S4014	MK978321	34	38, 54		4	Jinhua, Zhejiang	[34]	[34]
C. tanakae	S4038	MK765787	17			4	Jinhua, Zhejiang	[30]	
C. tanakae	S4050	MK765788	34			4	Jinhua, Zhejiang	[30]	
C. tanakae	S4060	MK765789	46		Yes	4	Jinhua, Zhejiang	[30]	
C. tanakae	S4066	MK978322	34	40, 56		4	Jinhua, Zhejiang	[34]	[34]
C. tanakae	S4067	MK978323	51	39, 54		4	Jinhua, Zhejiang	[34]	[34]
C. tanakae	S4068	MK978324	34	40, Uncertain		4	Jinhua, Zhejiang	[34]	[34]
C. tanakae	S4069	MK978325	17	39, 56		4	Jinhua, Zhejiang	[34]	[34]
C. tanakae	S4184	OP594789	34			4	Jinhua, Zhejiang	Current study	
C. tanakae	S4187	OP594790	34			4	Jinhua, Zhejiang	Current study	
C. tanakae	S4192	OP594791	46			4	Jinhua, Zhejiang	Current study	
C. tanakae	S4193	OP594792	17			4	Jinhua, Zhejiang	Current study	
C. tanakae	S4201	OP594793	17			4	Jinhua, Zhejiang	Current study	
C. tanakae	S4202	OP594794	46			4	Jinhua, Zhejiang	Current	

						study
C. tanakae	S4220	OP594795	34	4	Jinhua, Zhejiang	Current study
C. tanakae	S4333	OP594796	34	4	Jinhua, Zhejiang	Current study
C. tanakae	S4335	OP594797	17	4	Jinhua, Zhejiang	Current study
C. tanakae	S4337	OP594798	34	4	Jinhua, Zhejiang	Current study
C. tanakae	S4339	OP594799	34	4	Jinhua, Zhejiang	Current study
C. tanakae	S4377	OP594800	48	7	Jinggangshan, Jiangxi	Current study
C. tanakae	S4387	OP594801	52	8	Meihua, Shaoguan	Current study
C. tanakae	S4860	OP594802	36	16	Nanyang, Henan	Current study
C. tanakae		MN690963	63	22	Mt. Fanjing, Guizhou	[16]
C. tanakae		MN690967	55	21	Chongzuo, Guangxi	[16]
C. tanakae		MN690968	62	21	Chongzuo, Guangxi	[16]
C. tanakae		MN690977	59	25	Hejiang, Sichuan	[16]
C. tanakae		MN690964	33	22	Mt. Fanjing, Guizhou	[16]
C. tanakae		MN690965	40	21	Chongzuo, Guangxi	[16]
C. tanakae		MN690966	6	D1	Liangzhong, Sichuan	[16]
C. tanakae		MN690969	58	D2	Hongjiang, Hunan	[16]
C. tanakae		MN690970	56	D2	Hongjiang, Hunan	[16]
C. tanakae		MN690971	58	D2	Hongjiang, Hunan	[16]
C. tanakae		MN690972	17	D3	Mt. Yuelushan, Hunan	[16]

C. tanakae	MN690973	17	D4	Mt. Hengshan, Hunan	[16]
C. tanakae	MN690974	64	D3	Mt. Yuelushan, Hunan	[16]
C. tanakae	MN690975	61	D4	Mt. Hengshan, Hunan	[16]
C. tanakae	MN690976	17	D4	Mt. Hengshan, Hunan	[16]
C. tanakae	MN690978	40	25	Hejiang, Sichuan	[16]
C. tanakae	MN690979	60	D3	Mt. Yuelushan, Hunan	[16]
C. tanakae	MN690980	40	A7	Mt. Huaying, Sichuan	[16]
C. tanakae	MN690981	40	A7	Mt. Huaying, Sichuan	[16]
C. tanakae	MN690982	40	A7	Mt. Huaying, Sichuan	[16]
C. tanakae	MN690983	40	D5	Santai, Sichuan	[16]
C. tanakae	MN690984	17	C2	Dongyang, Zhejiang	[16]
C. tanakae	MN690985	53	C2	Dongyang, Zhejiang	[16]
C. tanakae	MN690986	34	C2	Dongyang, Zhejiang	[16]
C. tanakae	MN690987	34	C2	Dongyang, Zhejiang	[16]
C. tanakae	MN690988	54	C2	Dongyang, Zhejiang	[16]
C. tanakae	MN690989	40	C2	Dongyang, Zhejiang	[16]
C. tanakae	MN690990	40	C2	Dongyang, Zhejiang	[16]
C. tanakae	MN690991	27	26	Mt. Emei, Sichuan	[16]
C. tanakae	MN690992	57	22	Shiqian, Guizhou	[16]
C. tanakae	MN690993	27	26	Mt. Emei, Sichuan	[16]
C. tanakae	MK881609	17	B1	Mt. Huang, Anhui	[56]
C. tanakae	MK881610	17	B1	Mt. Huang, Anhui	[56]
C. tanakae	MK881611	67	D6	Xuancheng, Anhui	[56]
C. tanakae	MH429124	17	3	Xingshan, Hubei	[57]
C. tanakae	MH429125	65	3	Xingshan, Hubei	[57]
C. tanakae	MH429126	66	3	Xingshan, Hubei	[57]

C. tanakae	MN812267	68	D7	Pengze, Jiangxi	[58]
C. tanakae	KX946002	57	22	Mt. Fanjing, Guizhou	[59]
C. tanakae	KX946003	40	D8	Dujiangyan, Sichuan	[59]
C. tanakae	KX946004	40	D8	Dujiangyan, Sichuan	[59]
C. tanakae	KX946005	27	26	Mt. Emei, Sichuan	[59]
C. tanakae	KX946006	27	26	Mt. Emei, Sichuan	[59]
C. tanakae	FJ814035	69	D3	Liuyang, Hunan	[27]
C. tanakae	FJ814037	36	D3	Liuyang, Hunan	[27]
C. tanakae	FJ814038	70	D3	Liuyang, Hunan	[27]
C. tanakae	FJ814044	55	D9	Napo, Guangxi	[27]
C. tanakae	FJ814045	71	D10	Dongxing, Guangxi	[27]
C. tanakae	FJ814046	72	D10	Dongxing, Guangxi	[27]
C. tanakae	FJ814047	73	D10	Dongxing, Guangxi	[27]
C. tanakae	GU358528	74	D11	Taoyuan, Taiwan	[27]
C. tanakae	GU358529	75	D12	Taizhong, Taiwan	[27]
C. tanakae	GU358530	75	D12	Taizhong, Taiwan	[27]
C. tanakae	GU358531	76	D12	Taizhong, Taiwan	[27]
C. tanakae	GU358532	77	D13	Nantou, Taiwan	[27]
C. tanakae	AB175080	76	D13	Nantou, Taiwan	[25]
C. tanakae	AB175081	76	D13	Nantou, Taiwan	[25]
C.sp	FJ814039	101		Guangxi	[26]
