# Supplementary material for: The Effectiveness of Molecular, Karyotype and Morphological Methods in the Identification of Morphologically Conservative Sibling Species: An Integrative Taxonomic Case of the Crocidura attenuata Species Complex in Mainland China
Source: Animals (Basel). 2023 Feb 12;13(4):643. doi: 10.3390/ani13040643 (PMC9951653; doi:10.3390/ani13040643)
Supplement: Supplementary file 1 [file animals-13-00643-s001.zip › Table S2.pdf]

Table S2. Karyotype of the *C. attenuata* species complex and other reported *Crocidura* species in China. Species name, karyotype (2n, diploid chromosome number; FN, fundamental number including two X chromosomes; X, X chromosome; Y, Y chromosome; M, metacentric; SM, submetacentric; ST, subtelocentric; A, acrocentric; chromosome terminology following Maddalena and Ruedi [64]).

**Species**	**Karyotype**				**References**
	**2n**	**FN**	**X**	**Y**	
C. tanakae	24-40	45-56	SM, A	A	[34]
C. dongyangjiangensis	40	54	SM	A	In this study
C. anhuiensis	40	54	SM	A	[34]
C. attenuata	40	54	SM	A	[34]
C. Shantungensis	40	50	SM	A	[60]
C. lasiura	40	54	M	A	[61]
C. fuliginosa	40	54-58	SM	SM, A	[62]
C. sibirica	40	50	M	ST	[63]
