# Supplementary material for: The Effectiveness of Molecular, Karyotype and Morphological Methods in the Identification of Morphologically Conservative Sibling Species: An Integrative Taxonomic Case of the Crocidura attenuata Species Complex in Mainland China
Source: Animals (Basel). 2023 Feb 12;13(4):643. doi: 10.3390/ani13040643 (PMC9951653; doi:10.3390/ani13040643)
Supplement: Supplementary file 1 [file animals-13-00643-s001.zip › Table S3.pdf]

Table S3. One-way ANOVA among four species of *C. attenuata* species complex.

Index	F-value	df	Significance
HB	11.07039547	3, 60	P<0.001
Tail	8.586619377	3, 59	P<0.001
Tail/HB	3.855475187	3, 59	P<0.050
Ear	1.743615746	3, 60	0.1677
HF	40.41538239	3, 61	P<0.001
CIL	55.77235794	3, 54	P<0.001
MTR	97.58605194	3, 61	P<0.001
HCC)	22.5689888	3, 57	P<0.001
RW	37.21092224	3, 61	P<0.001
MB	31.06727219	3, 61	P<0.001
IO	48.91973877	3, 59	P<0.001
GW	51.57899094	3, 57	P<0.001
PIL	64.64163208	3, 59	P<0.001
PAL	57.82767105	3, 59	P<0.001
PPL	40.29610443	3, 54	P<0.001
UTR	71.87400818	3, 60	P<0.001
P^4-M^3	73.10980988	3, 61	P<0.001
PW1	48.91878891	3, 59	P<0.001
PGL	40.80350113	3, 59	P<0.001
LDT2	78.96796417	3, 61	P<0.001
LDT1	46.58351898	3, 61	P<0.001
M_1-M_3	61.14859009	3, 61	P<0.001
BCP	17.7464695	3, 61	P<0.001
ML	71.43994141	3, 60	P<0.001
COR	48.18684387	3, 61	P<0.001

Note: HB: head and body length, Tail: tail length, Tail/HB: tail length/head and body length, Ear: ear length, HF: hind foot length, CIL: condylo-incisive length, HCC: height of cranial capsule, RW: rostrum width, MB: maxillary breadth, IO: least interorbital width, GW: greatest width of skull, UTR: upper toothrow length, P^4–M^3: length of anterior tip of P^4 to posterior border of M^3, b PW1: readth of palate between the buccal margins of second molars, PGL: postglenoid width, M^1–M^3:length of lower molar series, ML: length of mandible from tip of incisor to posterior edge of condyle, COR: height of coronoid process, MTR: length of maxillary tooth row, PAL: palatilar length, PPL: post-palatal length, LDT1: length of dentary teeth excluding incisors, LDT2: length of dentary teeth including incisors, PIL: palato-incisor length and BCP: breadth of coronoid process.
