# Supplementary material for: The Effectiveness of Molecular, Karyotype and Morphological Methods in the Identification of Morphologically Conservative Sibling Species: An Integrative Taxonomic Case of the Crocidura attenuata Species Complex in Mainland China
Source: Animals (Basel). 2023 Feb 12;13(4):643. doi: 10.3390/ani13040643 (PMC9951653; doi:10.3390/ani13040643)
Supplement: Supplementary file 1 [file animals-13-00643-s001.zip › Table S4.pdf]

Table S4. The interspecific comparison of morphological measurement indexes among four species of *C. attenuata* species complex.

Index	Species 1 vs. Species 2	Mean±SD (n)	Mean±SD (n)	Diff.	$LSD_{0.05}$	Sig.
HB	*C.attenuata-C.tanakae*	74.88±6.54(16)	76.31±6.6(29)	1.43	4.133	ns
	C.attenuata-C.anhuiensis	74.88±6.54(16)	75.75±3.95(8)	0.87	5.246	ns
	C.attenuata-C.dongYJ	74.88±6.54(16)	63.82±6.54(11)	11.06	5.275	*
	C.tanakae-C.anhuiensis	76.31±6.6(29)	75.75±3.95(8)	0.56	4.996	ns
	C.tanakae-C.dongYJ	76.31±6.6(29)	63.82±6.54(11)	12.49	4.720	*
	C.anhuiensis-C.dongYJ	75.75±3.95(8)	63.82±6.54(11)	11.93	5.505	*
Tail	*C.attenuata-C.tanakae*	56.56±4.8(16)	52.29±5.66(28)	4.27	3.394	*
	C.attenuata-C.anhuiensis	56.56±4.8(16)	58.75±5.87(8)	2.19	4.638	ns
	C.attenuata-C.dongYJ	56.56±4.8(16)	48.32±4.11(11)	8.24	3.662	*
	C.tanakae-C.anhuiensis	52.29±5.66(28)	58.75±5.87(8)	6.46	4.644	*
	C.tanakae-C.dongYJ	52.29±5.66(28)	48.32±4.11(11)	3.97	3.809	*
	C.anhuiensis-C.dongYJ	58.75±5.87(8)	48.32±4.11(11)	10.43	4.815	*
Tail/HB	*C.attenuata-C.tanakae*	0.76±0.09(16)	0.69±0.09(28)	0.07	0.057	*
	C.attenuata-C.anhuiensis	0.76±0.09(16)	0.78±0.08(8)	0.02	0.078	ns
	C.attenuata-C.dongYJ	0.76±0.09(16)	0.76±0.07(11)	0.00	0.067	ns
	C.tanakae-C.anhuiensis	0.69±0.09(28)	0.78±0.08(8)	0.09	0.072	*
	C.tanakae-C.dongYJ	0.69±0.09(28)	0.76±0.07(11)	0.07	0.062	*
	C.anhuiensis-C.dongYJ	0.78±0.08(8)	0.76±0.07(11)	0.02	0.075	ns
HF	*C.attenuata-C.tanakae*	14.19±1.1(16)	12.98±0.66(30)	1.21	0.520	*
	C.attenuata-C.anhuiensis	14.19±1.1(16)	14.76±0.71(8)	0.57	0.891	ns
	C.attenuata-C.dongYJ	14.19±1.1(16)	11.39±0.5(11)	2.8	0.733	*
	C.tanakae-C.anhuiensis	12.98±0.66(30)	14.76±0.71(8)	1.78	0.538	*
	C.tanakae-C.dongYJ	12.98±0.66(30)	11.39±0.5(11)	1.59	0.441	*
	C.anhuiensis-C.dongYJ	14.76±0.71(8)	11.39±0.5(11)	3.37	0.583	*

CIL	*C.attenuata-C.tanakae*	21.06±0.56(14)	20.2±0.64(30)	0.86	0.405	*
	C.attenuata-C.anhuiensis	21.06±0.56(14)	21.34±0.77(7)	0.28	0.617	ns
	C.attenuata-C.dongYJ	21.06±0.56(14)	17.37±0.88(7)	3.69	0.660	*
	C.tanakae-C.anhuiensis	20.20±0.64(30)	21.34±0.77(7)	1.14	0.568	*
	C.tanakae-C.dongYJ	20.20±0.64(30)	17.37±0.88(7)	2.83	0.588	*
	C.anhuiensis-C.dongYJ	21.34±0.77(7)	17.37±0.88(7)	3.97	0.966	*
MTR	*C.attenuata-C.tanakae*	5.88±0.12(16)	5.82±0.15(30)	0.06	0.088	ns
	C.attenuata-C.anhuiensis	5.88±0.12(16)	6.25±0.22(8)	0.37	0.139	*
	C.attenuata-C.dongYJ	5.88±0.12(16)	4.89±0.32(11)	0.99	0.177	*
	C.tanakae-C.anhuiensis	5.82±0.15(30)	6.25±0.22(8)	0.43	0.135	*
	C.tanakae-C.dongYJ	5.82±0.15(30)	4.89±0.32(11)	0.93	0.148	*
	C.anhuiensis-C.dongYJ	6.25±0.22(8)	4.89±0.32(11)	1.36	0.273	*
HCC	*C.attenuata-C.tanakae*	5.03±0.14(15)	4.96±0.2(30)	0.07	0.115	ns
	C.attenuata-C.anhuiensis	5.03±0.14(15)	5.15±0.16(8)	0.12	0.132	ns
	C.attenuata-C.dongYJ	5.03±0.14(15)	4.42±0.3(8)	0.61	0.188	*
	C.tanakae-C.anhuiensis	4.96±0.2(30)	5.15±0.16(8)	0.19	0.153	*
	C.tanakae-C.dongYJ	4.96±0.2(30)	4.42±0.3(8)	0.54	0.178	*
	C.anhuiensis-C.dongYJ	5.15±0.16(8)	4.42±0.3(8)	0.73	0.256	*
RW	*C.attenuata-C.tanakae*	2.30±0.16(16)	2.48±0.14(30)	0.18	0.090	*
	C.attenuata-C.anhuiensis	2.30±0.16(16)	2.62±0.15(8)	0.32	0.139	*
	C.attenuata-C.dongYJ	2.30±0.16(16)	2.02±0.15(11)	0.28	0.124	*
	C.tanakae-C.anhuiensis	2.48±0.14(30)	2.62±0.15(8)	0.14	0.111	*
	C.tanakae-C.dongYJ	2.48±0.14(30)	2.02±0.15(11)	0.46	0.098	*
	C.anhuiensis-C.dongYJ	2.62±0.15(8)	2.02±0.15(11)	0.60	0.143	*
MB	*C.attenuata-C.tanakae*	6.47±0.28(16)	6.64±0.47(30)	0.17	0.259	ns
	C.attenuata-C.anhuiensis	6.47±0.28(16)	7.02±0.35(8)	0.55	0.275	*
	C.attenuata-C.dongYJ	6.47±0.28(16)	5.46±0.33(11)	1.01	0.244	*
	C.tanakae-C.anhuiensis	6.64±0.47(30)	7.02±0.35(8)	0.38	0.360	*
	C.tanakae-C.dongYJ	6.64±0.47(30)	5.46±0.33(11)	1.18	0.311	*
	C.anhuiensis-C.dongYJ	7.02±0.35(8)	5.46±0.33(11)	1.56	0.329	*
IO	*C.attenuata-C.tanakae*	4.55±0.15(15)	4.43±0.16(30)	0.12	0.101	*
	C.attenuata-C.anhuiensis	4.55±0.15(15)	4.90±0.24(8)	0.35	0.166	*
	C.attenuata-C.dongYJ	4.55±0.15(15)	3.92±0.2(10)	0.63	0.143	*
	C.tanakae-C.anhuiensis	4.43±0.16(30)	4.90±0.24(8)	0.47	0.145	*

	C.tanakae-C.dongYJ	4.43±0.16(30)	3.92±0.2(10)	0.51	0.127	*
	C.anhuiensis-C.dongYJ	4.90±0.24(8)	3.92±0.2(10)	0.98	0.216	*
GW	*C.attenuata-C.tanakae*	9.45±0.29(15)	9.13±0.3(30)	0.32	0.187	*
	C.attenuata-C.anhuiensis	9.45±0.29(15)	9.80±0.4(7)	0.35	0.310	*
	C.attenuata-C.dongYJ	9.45±0.29(15)	8.04±0.36(9)	1.41	0.275	*
	C.tanakae-C.anhuiensis	9.13±0.3(30)	9.80±0.4(7)	0.67	0.269	*
	C.tanakae-C.dongYJ	9.13±0.3(30)	8.04±0.36(9)	1.09	0.238	*
	C.anhuiensis-C.dongYJ	9.80±0.4(7)	8.04±0.36(9)	1.76	0.404	*
PIL	*C.attenuata-C.tanakae*	9.56±0.3(16)	9.28±0.3(30)	0.28	0.185	*
	C.attenuata-C.anhuiensis	9.56±0.3(16)	9.89±0.5(7)	0.33	0.345	ns
	C.attenuata-C.dongYJ	9.56±0.3(16)	7.71±0.58(10)	1.85	0.355	*
	C.tanakae-C.anhuiensis	9.28±0.3(30)	9.89±0.5(7)	0.61	0.291	*
	C.tanakae-C.dongYJ	9.28±0.3(30)	7.71±0.58(10)	1.57	0.284	*
	C.anhuiensis-C.dongYJ	9.89±0.5(7)	7.71±0.58(10)	2.18	0.580	*
PAL	*C.attenuata-C.tanakae*	7.99±0.26(16)	7.83±0.25(30)	0.16	0.161	ns
	C.attenuata-C.anhuiensis	7.99±0.26(16)	8.36±0.37(8)	0.37	0.271	*
	C.attenuata-C.dongYJ	7.99±0.26(16)	6.56±0.48(9)	1.43	0.305	*
	C.tanakae-C.anhuiensis	7.83±0.25(30)	8.36±0.37(8)	0.53	0.226	*
	C.tanakae-C.dongYJ	7.83±0.25(30)	6.56±0.48(9)	1.27	0.243	*
	C.anhuiensis-C.dongYJ	8.36±0.37(8)	6.56±0.48(9)	1.8	0.446	*
PPL	*C.attenuata-C.tanakae*	9.45±0.31(13)	8.94±0.4(30)	0.51	0.254	*
	C.attenuata-C.anhuiensis	9.45±0.31(13)	9.50±0.37(8)	0.05	0.315	ns
	C.attenuata-C.dongYJ	9.45±0.31(13)	7.72±0.22(7)	1.73	0.280	*
	C.tanakae-C.anhuiensis	8.94±0.4(30)	9.50±0.37(8)	0.56	0.322	*
	C.tanakae-C.dongYJ	8.94±0.4(30)	7.72±0.22(7)	1.22	0.324	*
	C.anhuiensis-C.dongYJ	9.50±0.37(8)	7.72±0.22(7)	1.78	0.350	*
UTR	*C.attenuata-C.tanakae*	9.21±0.24(16)	8.93±0.29(30)	0.28	0.170	*
	C.attenuata-C.anhuiensis	9.21±0.24(16)	9.61±0.42(7)	0.4	0.283	*
	C.attenuata-C.dongYJ	9.21±0.24(16)	7.61±0.46(11)	1.6	0.280	*
	C.tanakae-C.anhuiensis	8.93±0.29(30)	9.61±0.42(7)	0.68	0.267	*
	C.tanakae-C.dongYJ	8.93±0.29(30)	7.61±0.46(11)	1.32	0.244	*
	C.anhuiensis-C.dongYJ	9.61±0.42(7)	7.61±0.46(11)	2	0.457	*
P^4-M^3	*C.attenuata-C.tanakae*	5.16±0.12(16)	5.12±0.15(30)	0.04	0.087	ns
	C.attenuata-C.anhuiensis	5.16±0.12(16)	5.57±0.22(8)	0.41	0.142	*
	C.attenuata-C.dongYJ	5.16±0.12(16)	4.4±0.28(11)	0.76	0.164	*
	C.tanakae-C.anhuiensis	5.12±0.15(30)	5.57±0.22(8)	0.45	0.133	*
	C.tanakae-C.dongYJ	5.12±0.15(30)	4.4±0.28(11)	0.72	0.138	*
	C.anhuiensis-C.dongYJ	5.57±0.22(8)	4.4±0.28(11)	1.17	0.254	*

PW1	*C.attenuata-C.tanakae*	6.19±0.2(15)	6.17±0.25(30)	0.02	0.151	ns
	C.attenuata-C.anhuiensis	6.19±0.2(15)	6.59±0.28(8)	0.4	0.210	*
	C.attenuata-C.dongYJ	6.19±0.2(15)	5.23±0.33(10)	0.96	0.217	*
	C.tanakae-C.anhuiensis	6.17±0.25(30)	6.59±0.28(8)	0.42	0.208	*
	C.tanakae-C.dongYJ	6.17±0.25(30)	5.23±0.33(10)	0.94	0.200	*
	C.anhuiensis-C.dongYJ	6.59±0.28(8)	5.23±0.33(10)	1.36	0.309	*
PGL	*C.attenuata-C.tanakae*	6.48±0.16(15)	6.42±0.23(30)	0.06	0.136	ns
	C.attenuata-C.anhuiensis	6.48±0.16(15)	6.80±0.34(8)	0.32	0.215	*
	C.attenuata-C.dongYJ	6.48±0.16(15)	5.57±0.35(10)	0.91	0.214	*
	C.tanakae-C.anhuiensis	6.42±0.23(30)	6.80±0.34(8)	0.38	0.209	*
	C.tanakae-C.dongYJ	6.42±0.23(30)	5.57±0.35(10)	0.85	0.197	*
	C.anhuiensis-C.dongYJ	6.80±0.34(8)	5.57±0.35(10)	1.23	0.349	*
LDT2	*C.attenuata-C.tanakae*	8.47±0.19(16)	8.18±0.27(30)	0.29	0.155	*
	C.attenuata-C.anhuiensis	8.47±0.19(16)	8.86±0.41(8)	0.39	0.250	*
	C.attenuata-C.dongYJ	8.47±0.19(16)	6.92±0.43(11)	1.55	0.250	*
	C.tanakae-C.anhuiensis	8.18±0.27(30)	8.86±0.41(8)	0.68	0.246	*
	C.tanakae-C.dongYJ	8.18±0.27(30)	6.92±0.43(11)	1.26	0.230	*
	C.anhuiensis-C.dongYJ	8.86±0.41(8)	6.92±0.43(11)	1.94	0.414	*
LDT1	*C.attenuata-C.tanakae*	6.20±0.14(16)	6.06±0.22(30)	0.14	0.124	*
	C.attenuata-C.anhuiensis	6.20±0.14(16)	6.53±0.44(8)	0.33	0.246	*
	C.attenuata-C.dongYJ	6.20±0.14(16)	5.24±0.31(11)	0.96	0.183	*
	C.tanakae-C.anhuiensis	6.06±0.22(30)	6.53±0.44(8)	0.47	0.225	*
	C.tanakae-C.dongYJ	6.06±0.22(30)	5.24±0.31(11)	0.82	0.178	*
	C.anhuiensis-C.dongYJ	6.53±0.44(8)	5.24±0.31(11)	1.29	0.363	*
M_1-M_3	*C.attenuata-C.tanakae*	4.24±0.11(16)	4.19±0.14(30)	0.05	0.081	ns
	C.attenuata-C.anhuiensis	4.24±0.11(16)	4.56±0.18(8)	0.32	0.119	*
	C.attenuata-C.dongYJ	4.24±0.11(16)	3.65±0.22(11)	0.59	0.129	*
	C.tanakae-C.anhuiensis	4.19±0.14(30)	4.56±0.18(8)	0.37	0.119	*
	C.tanakae-C.dongYJ	4.19±0.14(30)	3.65±0.22(11)	0.54	0.116	*
	C.anhuiensis-C.dongYJ	4.56±0.18(8)	3.65±0.22(11)	0.91	0.197	*
BCP	*C.attenuata-C.tanakae*	1.00±0.10(16)	1.07±0.1(30)	0.07	0.062	*
	C.attenuata-C.anhuiensis	1.00±0.10(16)	1.16±0.10(8)	0.16	0.091	*
	C.attenuata-C.dongYJ	1.00±0.10(16)	0.86±0.09(11)	0.14	0.077	*
	C.tanakae-C.anhuiensis	1.07±0.10(30)	1.16±0.10(8)	0.09	0.081	*
	C.tanakae-C.dongYJ	1.07±0.10(30)	0.86±0.09(11)	0.21	0.069	*
	C.anhuiensis-C.dongYJ	1.16±0.10(8)	0.86±0.09(11)	0.3	0.094	*
ML	*C.attenuata-C.tanakae*	13.23±0.41(16)	12.68±0.43(30)	0.55	0.263	*

	C.attenuata-C.anhuiensis	13.23±0.41(16)	13.78±0.70(8)	0.55	0.469	*
	C.attenuata-C.dongYJ	13.23±0.41(16)	10.7±0.64(10)	2.53	0.424	*
	C.tanakae-C.anhuiensis	12.68±0.43(30)	13.78±0.70(8)	1.1	0.397	*
	C.tanakae-C.dongYJ	12.68±0.43(30)	10.70±0.64(10)	1.98	0.359	*
	C.anhuiensis-C.dongYJ	13.78±0.70(8)	10.70±0.64(10)	3.08	0.673	*
COR	*C.attenuata-C.tanakae*	4.90±0.2(16)	4.84±0.23(30)	0.06	0.137	ns
	C.attenuata-C.anhuiensis	4.90±0.2(16)	5.29±0.30(8)	0.39	0.213	*
	C.attenuata-C.dongYJ	4.90±0.2(16)	4.06±0.26(11)	0.84	0.181	*
	C.tanakae-C.anhuiensis	4.84±0.23(30)	5.29±0.30(8)	0.45	0.198	*
	C.tanakae-C.dongYJ	4.84±0.23(30)	4.06±0.26(11)	0.78	0.169	*
	C.anhuiensis-C.dongYJ	5.29±0.3(8)	4.06±0.26(11)	1.23	0.271	*

Note: *C. dongYJ* is for *C. dongyangjiang*. HB: head and body length, Tail: tail length, Tail/HB: tail length/head and body length, Ear: ear length, HF: hind foot length, CIL: condylo-incisive length, HCC: height of cranial capsule, RW: rostrum width, MB: maxillary breadth, IO: least interorbital width, GW: greatest width of skull, UTR: upper toothrow length, P^4–M^3: length of anterior tip of P^4 to posterior border of M^3, b PW1: readth of palate between the buccal margins of second molars, PGL: postglenoid width, M^1–M^3:length of lower molar series, ML: length of mandible from tip of incisor to posterior edge of condyle, COR: height of coronoid process, MTR: length of maxillary tooth row, PAL: palatilar length, PPL: post-palatal length, LDT1: length of dentary teeth excluding incisors, LDT2: length of dentary teeth including incisors, PIL: palato-incisor length and BCP: breadth of coronoid process.
