# Supplementary figures and images for: The Effectiveness of Molecular, Karyotype and Morphological Methods in the Identification of Morphologically Conservative Sibling Species: An Integrative Taxonomic Case of the Crocidura attenuata Species Complex in Mainland China
Source: Animals (Basel). 2023 Feb 12;13(4):643. doi: 10.3390/ani13040643 (PMC9951653; doi:10.3390/ani13040643)

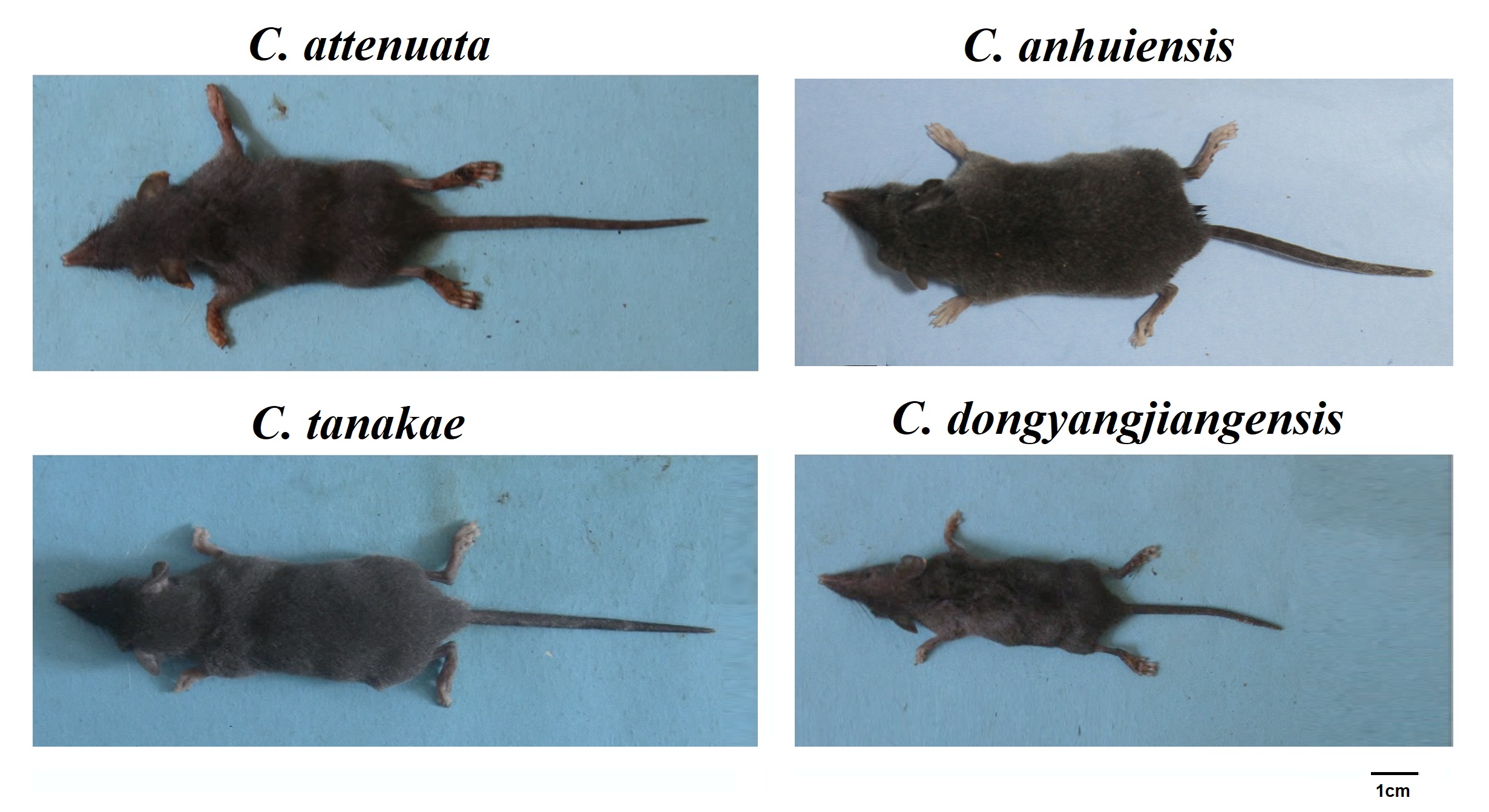

Supplement: Supplementary file 1 [file animals-13-00643-s001.zip › Figure S1.jpg]

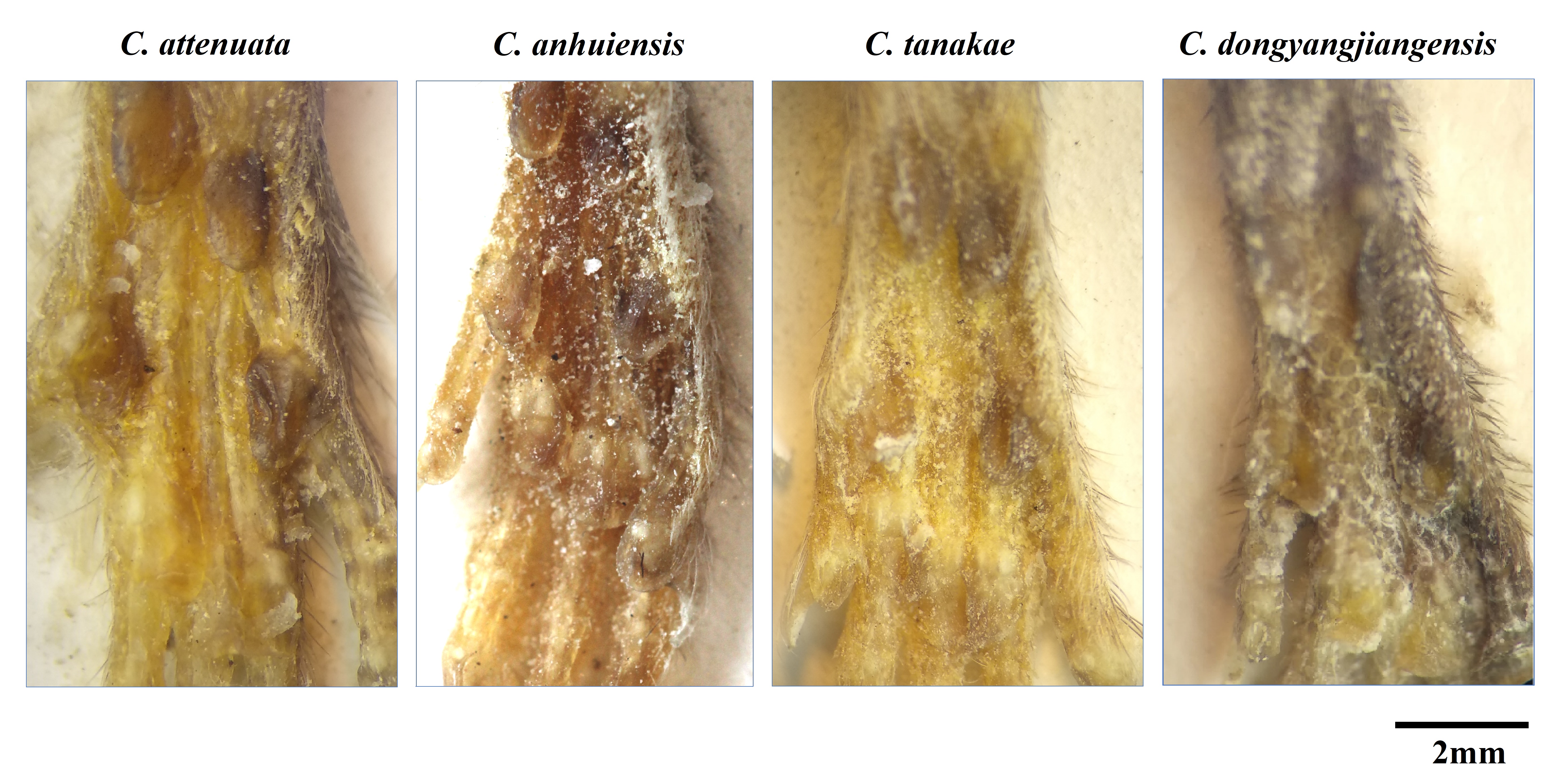

Supplement: Supplementary file 1 [file animals-13-00643-s001.zip › Figure S2.jpg]
